# Supplementary material for: Machine Learning Accelerates Crystallization for Structure Determination
Source: Angew Chem Int Ed Engl. 2026 May 4;65(25):e1218503. doi: 10.1002/anie.1218503 (PMC13266958; doi:10.1002/anie.1218503)

checkCIF/PLATON report

Structure factors have been supplied for datablock(s) lcz042

THIS REPORT IS FOR GUIDANCE ONLY. IF USED AS PART OF A REVIEW PROCEDURE FOR PUBLICATION, IT SHOULD NOT REPLACE THE EXPERTISE OF AN EXPERIENCED CRYSTALLOGRAPHIC REFEREE.

No syntax errors found.   CIF dictionary   Interpreting this report

Datablock: lcz042

Bond precision:	C-C = 0.0187 A		Wavelength=1.54184
Cell:	a=14.2786(1)	b=25.1937(3)	c=21.4298(2)
	alpha=90	beta=91.691(1)	gamma=90
Temperature:	100 K		

	Calculated	Reported
Volume	7705.60(13)	7705.60(13)
Space group	P 21/n	P 1 21/n 1
Hall group	-P 2yn	-P 2yn
Moiety formula	C49 H35 Ag3 F18 N6 O6, C15 H3 Ag3 F18 N6	C15 H3 Ag3 F18 N6, C17 H16 O3
Sum formula	C64 H38 Ag6 F36 N12 O6	C32 H19 Ag3 F18 N6 O3
Mr	2402.29	1201.14
Dx,g cm-3	2.071	2.071
Z	4	8
Mu (mm-1)	13.331	13.331
F000	4640.0	4640.0
F000’	4661.71	
h,k,lmax	18,32,27	18,31,26
Nref	16568	15699
Tmin,Tmax	0.060,0.186	0.249,1.000
Tmin’	0.010	

Correction method= # Reported T Limits: Tmin=0.249 Tmax=1.000
AbsCorr = MULTI-SCAN

Data completeness= 0.948 Theta(max)= 78.572

R(reflections)= 0.1057(12545) wR2(reflections)= 0.2495(15699)

S = 1.090 Npar= 1067

The following ALERTS were generated. Each ALERT has the format
test-name_ALERT_alert-type_alert-level.
Click on the hyperlinks for more details of the test.

Alert level C

```
PLAT041_ALERT_1_C Calc. and Reported SumFormula    Strings  Differ     Please Check
              Calc: C64 H38 Ag6 F36 N12 O6
              Rep.: C32 H19 Ag3 F18 N6 O3
PLAT042_ALERT_1_C Calc. and Reported MoietyFormula Strings  Differ     Please Check
              Calc: C49 H35 Ag3 F18 N6 O6, C15 H3 Ag3 F18 N6
              Rep.: C15 H3 Ag3 F18 N6, C17 H16 O3
PLAT082_ALERT_2_C High R1 Value ..................................       0.11 Report
PLAT213_ALERT_2_C Atom F00H           has ADP max/min Ratio .....         3.2 prolat
PLAT213_ALERT_2_C Atom F01J           has ADP max/min Ratio .....         3.2 prolat
PLAT213_ALERT_2_C Atom F01Y           has ADP max/min Ratio .....         3.9 prolat
PLAT213_ALERT_2_C Atom F02J           has ADP max/min Ratio .....         3.4 prolat
PLAT213_ALERT_2_C Atom O00U           has ADP max/min Ratio .....         3.3 prolat
PLAT213_ALERT_2_C Atom O016           has ADP max/min Ratio .....         3.7 prolat
PLAT213_ALERT_2_C Atom C02B           has ADP max/min Ratio .....         3.3 prolat
PLAT214_ALERT_2_C Atom F00L      (Anion/Solvent) ADP max/min Ratio        4.6 prolat
PLAT220_ALERT_2_C NonSolvent   Resd 1  F   Ueq(max)/Ueq(min) Range        3.9 Ratio
PLAT230_ALERT_2_C Hirshfeld Test Diff for    F03H     --C035     .        6.8 s.u.
PLAT234_ALERT_4_C Large Hirshfeld Difference O00U     --C03B     .       0.18 Ang.
PLAT234_ALERT_4_C Large Hirshfeld Difference O016     --C038     .       0.17 Ang.
PLAT234_ALERT_4_C Large Hirshfeld Difference C01V     --C02B     .       0.19 Ang.
PLAT234_ALERT_4_C Large Hirshfeld Difference C010     --C02P     .       0.17 Ang.
PLAT234_ALERT_4_C Large Hirshfeld Difference C01X     --C021     .       0.18 Ang.
PLAT234_ALERT_4_C Large Hirshfeld Difference F009     --C02R     .       0.17 Ang.
PLAT234_ALERT_4_C Large Hirshfeld Difference F00L     --C029     .       0.17 Ang.
PLAT234_ALERT_4_C Large Hirshfeld Difference N00K     --N013     .       0.17 Ang.
PLAT242_ALERT_2_C Low    'MainMol' Ueq as Compared to Neighbors of       C022 Check
PLAT250_ALERT_2_C Large U3/U1 Ratio for <U(i,j)> Tensor(Resd    2)        2.2 Note
PLAT309_ALERT_2_C Single Bonded Oxygen (C-O > 1.3 Ang) ..........          O1 Check
PLAT342_ALERT_3_C Low Bond Precision on  C-C Bonds ...............    0.01866 Ang.
PLAT362_ALERT_2_C Short  C(sp3)-C(sp2) Bond  C02L     - C01R     .       1.41 Ang.
PLAT362_ALERT_2_C Short  C(sp3)-C(sp2) Bond  C034     - C032     .       1.41 Ang.
PLAT906_ALERT_3_C Large K Value in the Analysis of Variance ......     26.233 Check
PLAT906_ALERT_3_C Large K Value in the Analysis of Variance ......      4.687 Check
PLAT906_ALERT_3_C Large K Value in the Analysis of Variance ......      2.678 Check
PLAT911_ALERT_3_C Missing FCF Refl Between Thmin & STh/L=    0.600        159 Report
               17  2  0,   2 16  0,   2 20  0,   3 21  0, -17  0  1,  -1  0  1,
                1  0  1,  17  0  1,  17  1  1,  -5 23  1,   0 27  1,   0  0  2,
              -17  1  2,  17  1  2,  -5 23  2,  -4 23  2,  -6 28  2, -15  1  3,
              -15 12  3,   4 13  3,  -6 24  3,  -7 27  3,  15  6  4,  15  7  4,
               15  8  4,  -1  0  5,  15  7  5,  15  8  5,   3 13  5,  15  7  6,
                   (  129 More Missing: see the .ckf listing file)
PLAT971_ALERT_2_C Check Calcd Resid. Dens.  1.39Ang From F037            2.38 eA-3
```

```
PLAT971_ALERT_2_C Check Calcd Resid. Dens.  0.89Ang From Ag02           1.95 eA-3
PLAT971_ALERT_2_C Check Calcd Resid. Dens.  1.27Ang From Ag03           1.93 eA-3
PLAT971_ALERT_2_C Check Calcd Resid. Dens.  1.00Ang From F1             1.85 eA-3
PLAT971_ALERT_2_C Check Calcd Resid. Dens.  1.30Ang From Ag01           1.74 eA-3
PLAT971_ALERT_2_C Check Calcd Resid. Dens.  0.81Ang From Ag06           1.66 eA-3
PLAT971_ALERT_2_C Check Calcd Resid. Dens.  0.80Ang From Ag04           1.63 eA-3
PLAT971_ALERT_2_C Check Calcd Resid. Dens.  0.84Ang From N00E           1.61 eA-3
PLAT971_ALERT_2_C Check Calcd Resid. Dens.  0.77Ang From F037           1.60 eA-3
PLAT971_ALERT_2_C Check Calcd Resid. Dens.  0.97Ang From Ag03           1.58 eA-3
PLAT971_ALERT_2_C Check Calcd Resid. Dens.  1.20Ang From Ag03           1.56 eA-3
PLAT971_ALERT_2_C Check Calcd Resid. Dens.  1.35Ang From Ag02           1.51 eA-3
PLAT971_ALERT_2_C Check Calcd Resid. Dens.  1.19Ang From F01E           1.51 eA-3
PLAT972_ALERT_2_C Check Calcd Resid. Dens.  1.08Ang From Ag01          -2.16 eA-3
PLAT972_ALERT_2_C Check Calcd Resid. Dens.  0.86Ang From Ag03          -2.15 eA-3
PLAT972_ALERT_2_C Check Calcd Resid. Dens.  0.85Ang From Ag06          -1.99 eA-3
PLAT972_ALERT_2_C Check Calcd Resid. Dens.  0.94Ang From Ag03          -1.85 eA-3
PLAT972_ALERT_2_C Check Calcd Resid. Dens.  0.91Ang From Ag05          -1.74 eA-3
PLAT972_ALERT_2_C Check Calcd Resid. Dens.  0.94Ang From Ag03          -1.69 eA-3
PLAT972_ALERT_2_C Check Calcd Resid. Dens.  1.09Ang From Ag04          -1.65 eA-3
PLAT972_ALERT_2_C Check Calcd Resid. Dens.  0.85Ang From Ag04          -1.65 eA-3
PLAT972_ALERT_2_C Check Calcd Resid. Dens.  0.78Ang From Ag06          -1.63 eA-3
PLAT972_ALERT_2_C Check Calcd Resid. Dens.  1.39Ang From N00E          -1.62 eA-3
PLAT972_ALERT_2_C Check Calcd Resid. Dens.  1.10Ang From Ag03          -1.57 eA-3
PLAT972_ALERT_2_C Check Calcd Resid. Dens.  0.89Ang From Ag02          -1.53 eA-3
PLAT977_ALERT_2_C Check Negative Difference Density on H01S      .     -0.45 eA-3
PLAT977_ALERT_2_C Check Negative Difference Density on H021      .     -0.46 eA-3
PLAT977_ALERT_2_C Check Negative Difference Density on H027      .     -0.35 eA-3
PLAT977_ALERT_2_C Check Negative Difference Density on H02V      .     -0.47 eA-3
```

Alert level G

```
PLAT003_ALERT_2_G Number of Uiso or U(i,j)  Restrained non-H-Atoms        38 Report
PLAT045_ALERT_1_G Calculated and Reported Z Differ by a Factor ...     0.500 Check
PLAT083_ALERT_2_G SHELXL Second Parameter in WGHT  Unusually Large    469.86 Why ?
PLAT143_ALERT_4_G s.u. on c - Axis Small or Missing .............    0.00020 Ang.
PLAT171_ALERT_4_G The CIF-Embedded .res File Contains EADP Records         2 Report
PLAT177_ALERT_4_G The CIF-Embedded .res File Contains DELU Records         5 Report
PLAT178_ALERT_4_G The CIF-Embedded .res File Contains SIMU Records         4 Report
PLAT186_ALERT_4_G The CIF-Embedded .res File Contains ISOR Records         9 Report
PLAT188_ALERT_3_G A Non-default SIMU Restraint Value has been used    0.0200 Report
PLAT242_ALERT_2_G Low    'MainMol' Ueq as Compared to Neighbors of      C01R Check
PLAT242_ALERT_2_G Low    'MainMol' Ueq as Compared to Neighbors of      C02S Check
PLAT242_ALERT_2_G Low    'MainMol' Ueq as Compared to Neighbors of      C030 Check
PLAT242_ALERT_2_G Low    'MainMol' Ueq as Compared to Neighbors of      C032 Check
PLAT242_ALERT_2_G Low    'MainMol' Ueq as Compared to Neighbors of      C039 Check
PLAT242_ALERT_2_G Low    'MainMol' Ueq as Compared to Neighbors of      C03D Check
PLAT242_ALERT_2_G Low    'MainMol' Ueq as Compared to Neighbors of      C029 Check
PLAT242_ALERT_2_G Low    'MainMol' Ueq as Compared to Neighbors of      C02Z Check
PLAT242_ALERT_2_G Low    'MainMol' Ueq as Compared to Neighbors of      C033 Check
PLAT242_ALERT_2_G Low    'MainMol' Ueq as Compared to Neighbors of      C035 Check
PLAT242_ALERT_2_G Low    'MainMol' Ueq as Compared to Neighbors of      C036 Check
PLAT299_ALERT_4_G Atom Site Occupancy Constrained at ............        0.5 Check
              O1      O2      H021    H777
PLAT301_ALERT_3_G Main Residue  Disorder .............(Resd    1)         1% Note
PLAT431_ALERT_2_G Short Inter HL..A Contact  F1       ..O1       .      2.79 Ang.
                                         1-x,1-y,1-z  =     3_666 Check
PLAT434_ALERT_2_G Short Inter HL..HL Contact F01G     ..F037     .      2.70 Ang.
```

```
                                                 x,y,z  =      1_555 Check
PLAT720_ALERT_4_G Number of Unusual/Non-Standard Labels .........        159 Note
              Ag01    Ag02    Ag03    Ag04    Ag05    Ag06    F007    F008
              F009    F00A    F00B    F00C    F00D    N00E    F00F    F00G
              F00H    N00I    N00J    N00S    C02F    C01V    H01V    C02B
              N00K    F00L    F00M    F00N    F00O    O00Q    O00R    F00T
              O00U    F00V    F00W    F00X    O00Y    N00Z    C010    H010
              F011    N013    N015    O016    N017    N00P    C02H    C02A
              H02A    C02L    N018    N023    C03G    C02T    H02T    C034
              F019    C01K    C01U    H01U    C01T    H01T    C01A    C012
              H012    C014    H014    F01B    F01C    F01D    F01E    F01F
              F01G    C01H    F01I    F01J    C01L    H01L    C01M    C01N
              C01O    F01P    C01Q    C01R    C01S    H01S    C02K    H02K
              C02N    C02M    H02M    C025    H025    C020    C01X    F01Y
              C01Z    C021    H021    C022    C024    H024    C026    H026
              C027    H027    C028    H028    C029    C02C    H02C    C02D
              H02D    C02E    C02I    F02J    F02O    C02P    H02P    C02Q
              H02Q    C02R    C02S    C02U    H02U    C02V    H02V    C02W
              H02B    H02E    H02F    C02X    H02X    C02Z    C030    F031
              C032    C033    C035    C036    F037    C038    C039    C03A
              H03A    H03B    H03C    C03B    H03H    H03I    H03J    C03C
              H03D    C03D    C03F    H03E    H03F    H03G    F03H
PLAT860_ALERT_3_G Number of Least-Squares Restraints ............        265 Note
PLAT883_ALERT_1_G Absent Datum for _atom_sites_solution_primary ..     Please Do !
PLAT912_ALERT_4_G Missing # of FCF Reflections Above STh/L=  0.600       692 Note
PLAT933_ALERT_2_G Number of HKL-OMIT Records in Embedded .res File        35 Note
              -15  1  3, -12  3 15, -12  4 15, -11  0 19, -11  1 19, -11  6 14,
              -11  7 13,  -9  1 20,  -9  6 19,  -8  1 20,  -8  6 19,  -8  8 18,
               -7  1 19,  -7  1 20,  -7  2 19,  -7  3 19,  -7  4 19,  -7  7 18,
               -7  8 18,  -6  1 20,  -6  2 19,  -6  2 20,  -6  3 19,  -6  6 18,
               -5  1 19,  -5  1 20,  -5  2 18,  -5  2 19,  -5  2 20,  -5  3 20,
               -4  3 18,  -4  5 17,  -3  2 17,  -3  2 18,  -2  1 17,
PLAT941_ALERT_3_G Average HKL Measurement Multiplicity ..........        2.4 Low
PLAT969_ALERT_5_G The 'Henn et al.' R-Factor-gap value ..........      4.249 Note
              Predicted wR2: Based on SigI**2  5.87 or SHELX Weight 22.90
PLAT978_ALERT_2_G Number C-C Bonds with Positive Residual Density.         0 Info
```

```
   0 ALERT level A = Most likely a serious problem - resolve or explain
   0 ALERT level B = A potentially serious problem, consider carefully
  60 ALERT level C = Check. Ensure it is not caused by an omission or oversight
  32 ALERT level G = General information/check it is not something unexpected

   4 ALERT type 1 CIF construction/syntax error, inconsistent or missing data
  62 ALERT type 2 Indicator that the structure model may be wrong or deficient
   9 ALERT type 3 Indicator that the structure quality may be low
  16 ALERT type 4 Improvement, methodology, query or suggestion
   1 ALERT type 5 Informative message, check
```

Validation response form

Please find below a validation response form (VRF) that can be filled in and pasted into your CIF.

```
# start Validation Reply Form
_vrf_PLAT041_lcz042
;
PROBLEM: Calc. and Reported SumFormula    Strings  Differ     Please Check
RESPONSE: ...
;
_vrf_PLAT042_lcz042
;
PROBLEM: Calc. and Reported MoietyFormula Strings  Differ     Please Check
RESPONSE: ...
;
_vrf_PLAT082_lcz042
;
PROBLEM: High R1 Value ..................................       0.11 Report
RESPONSE: ...
;
_vrf_PLAT213_lcz042
;
PROBLEM: Atom F00H            has ADP max/min Ratio .....        3.2 prolat
RESPONSE: ...
;
_vrf_PLAT214_lcz042
;
PROBLEM: Atom F00L      (Anion/Solvent) ADP max/min Ratio        4.6 prolat
RESPONSE: ...
;
_vrf_PLAT220_lcz042
;
PROBLEM: NonSolvent   Resd 1   F   Ueq(max)/Ueq(min) Range        3.9 Ratio
RESPONSE: ...
;
_vrf_PLAT230_lcz042
;
PROBLEM: Hirshfeld Test Diff for    F03H     --C035     .        6.8 s.u.
RESPONSE: ...
;
_vrf_PLAT234_lcz042
;
PROBLEM: Large Hirshfeld Difference O00U     --C03B     .       0.18 Ang.
RESPONSE: ...
;
_vrf_PLAT242_lcz042
;
PROBLEM: Low    'MainMol' Ueq as Compared to Neighbors of       C022 Check
RESPONSE: ...
;
_vrf_PLAT250_lcz042
;
PROBLEM: Large U3/U1 Ratio for <U(i,j)> Tensor(Resd    2)        2.2 Note
RESPONSE: ...
;
_vrf_PLAT309_lcz042
;
PROBLEM: Single Bonded Oxygen (C-O > 1.3 Ang) ..........          O1 Check
RESPONSE: ...
;
_vrf_PLAT342_lcz042
```

```
;
PROBLEM: Low Bond Precision on  C-C Bonds ..............    0.01866 Ang.
RESPONSE: ...
;
_vrf_PLAT362_lcz042
;
PROBLEM: Short  C(sp3)-C(sp2) Bond  C02L     - C01R     .       1.41 Ang.
RESPONSE: ...
;
_vrf_PLAT906_lcz042
;
PROBLEM: Large K Value in the Analysis of Variance ......     26.233 Check
RESPONSE: ...
;
_vrf_PLAT911_lcz042
;
PROBLEM: Missing FCF Refl Between Thmin & STh/L=    0.600        159 Report
RESPONSE: ...
;
_vrf_PLAT971_lcz042
;
PROBLEM: Check Calcd Resid. Dens.  1.39Ang From F037            2.38 eA-3
RESPONSE: ...
;
_vrf_PLAT972_lcz042
;
PROBLEM: Check Calcd Resid. Dens.  1.08Ang From Ag01           -2.16 eA-3
RESPONSE: ...
;
_vrf_PLAT977_lcz042
;
PROBLEM: Check Negative Difference Density on H01S      .      -0.45 eA-3
RESPONSE: ...
;
# end Validation Reply Form
```

It is advisable to attempt to resolve as many as possible of the alerts in all categories. Often the minor alerts point to easily fixed oversights, errors and omissions in your CIF or refinement strategy, so attention to these fine details can be worthwhile. It is up to the individual to critically assess their own results and, if necessary, seek expert advice.

PLATON version of 17/09/2025; check.def file version of 17/09/2025

---

duplicate check

No duplication found

Datablock lcz042 - ellipsoid plot

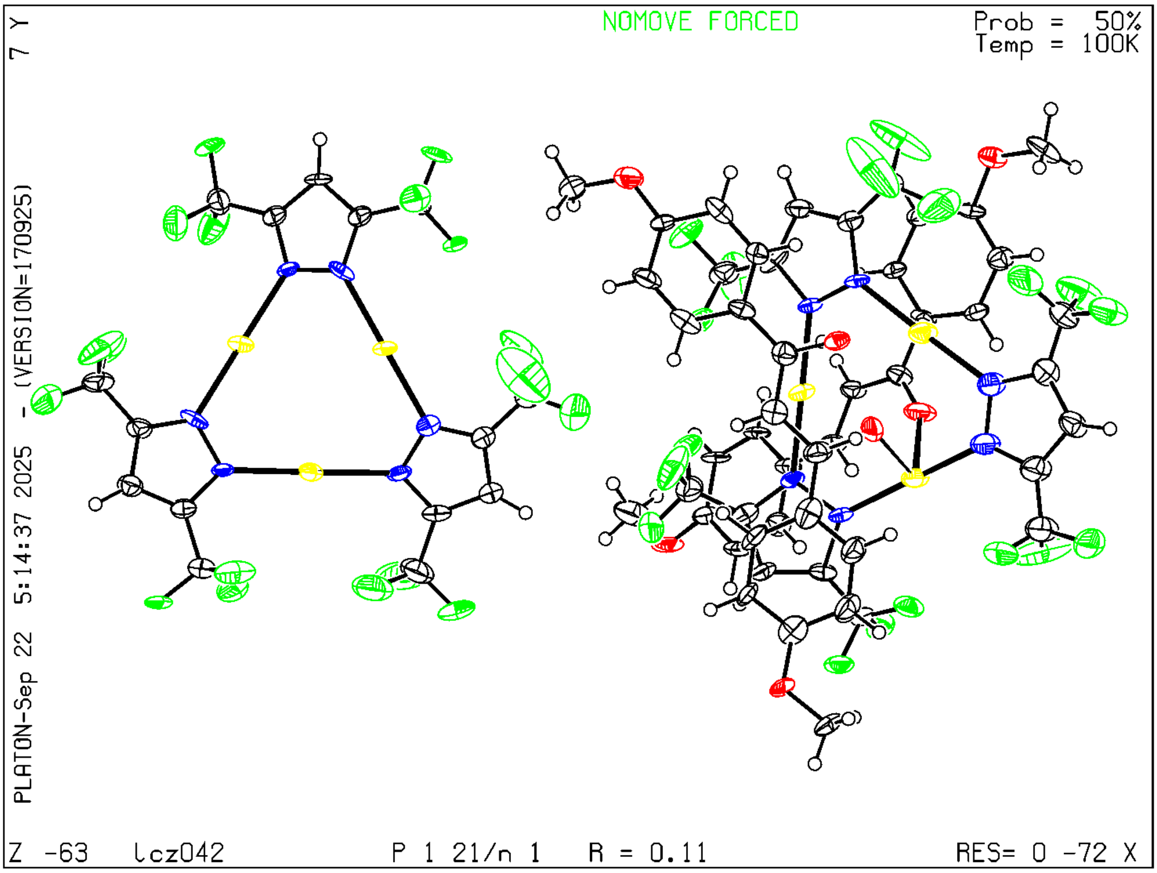

Supplement: Supplementary file 2 — Supporting File 2: anie72462‐sup‐0002‐DataFile.zip. [file ANIE-65-e1218503-s002.zip › supplementary_materials_crystallographic_data submit.zip!/cif_and_checkcif(1-114)/Ag3Pz3_42/Ag3Pz3_42_checkcif.pdf]
